# In vitro evaluation of the potential of aclarubicin in the treatment of small cell carcinoma of the lung (SCCL).

**DOI:** 10.1038/bjc.1989.376

**Published:** 1989-12

**Authors:** P. B. Jensen, L. VindelÃ¸v, H. Roed, E. J. Demant, M. Sehested, T. Skovsgaard, H. H. Hansen

**Affiliations:** Department of Oncology, Finsen Institute, Copenhagen, Denmark.

## Abstract

**Images:**


Br. J. Cancer (1989), 60, 838-844 © The Macmillan Press Ltd., 1989

### *In vitro* evaluation of the potential of aclarubicin in the treatment of small cell carcinoma of the lung (SCCL)

P.B. Jensen[1], L. Vindeløv[1,2,6], H. Roed[1], E.J.F. Demant[3], M. Sehested[4], T. Skovsgaard[5] & H.H. Hansen[1]

[1]*Department of Oncology, The Finsen Institute, 49 Strandboulevarden, DK-2100 Copenhagen Ø, Denmark;* [2]*Department of Internal Medicine, The Finsen Institute;* [3]*Department of Biochemistry C, University of Copenhagen Panum Institute, 3 Blegdamsvej DK-2100 Copenhagen Ø, Denmark;* [4]*Department of Pathology, Herlev University Hospital, DK-2730 Herlev, Denmark;* [5]*Department of Oncology, Herlev University Hospital;* [6]*Department of Internal Medicine C, Bispebjerg Hospital, Bispebjerg Bakke DK-2400 Copenhagen NV, Denmark.*

**Summary** The sensitivity of eight cell lines established from treated and untreated patients with small cell carcinoma of the lung (SCCL) was tested in the clonogenic assay with 1 h and continuous exposure to aclarubicin (ACLA), adriamycin (ADR), daunorubicin (DAU) and mitoxantrone (MITO). The sensitivity to ADR, DAU and MITO covariated, and varied with a factor of five. The sensitivity to ACLA was independent of the sensitivity to ADR and varied only within a factor of two. Only ACLA showed pronounced increased potency with continuous incubation, and ACLA was the most potent drug in the three cell lines least sensitive to ADR. Two resistant cell lines were selected by treating NCI-H69 *in vitro* with DAU. One cell line (9-fold resistant to DAU) expressed large amounts of P-glycoprotein, the other cell line (4-fold resistant to DAU) had barely detectable glycoprotein. Both lines acquired resistance to ADR, ACLA and MITO. The cross-resistance to ACLA and MITO was only partial and ACLA was still the most potent drug on these lines. The sensitivity to ACLA of the cell lines least sensitive to ADR suggest that ACLA partially circumvents mechanisms of multidrug resistance. Together with the pronounced increase in potency with prolonged exposure, these results suggest that ACLA has a mechanism of action different from the 'classical' anthracyclines. In this context mitoxantrone is more similar to the classical anthracyclines although its structure is more dissimilar.

The anthracyclines are among the most potent drugs in the treatment of a wide range of human neoplasms. Development of resistance to the drugs is a major problem, and much effort is devoted to the search of new anthracyclines without cross-resistance to the parent drugs. The anthracycline aclarubicin (ACLA) was isolated in 1975 by Oki *et al.* (1975) and activitiy was found in acute myelogenic leukaemia (AML) refractory to treatment with daunorubicin (DAU) and cytarbine (Pedersen-Bjergaard *et al.*, 1984; Warrel *et al.*, 1982). Recently, a phase III trial has found ACLA to be superior to DAU in the treatment of *de novo* AML (Hansen *et al.*, 1988). In a previous study on the inter-experimental variation in the clonogenic assay we observed that the sensitivity pattern to ACLA in a panel of small cell lung cancer (SCCL) cell lines was different from the sensitivity to adriamycin (ADR), DAU and mitoxantrone (MITO) after short time incubation (Jensen *et al.*, 1989). The present investigations were initiated to further elucidate the differences between ACLA and the other analogues. A murine tumour has recently been described to be relatively more sensitive to MITO than the tested human tumours (Hill *et al.*, 1989). The murine tumour cells P388 and Ehrlich were included in the present study for comparision to the human cell lines and the *in vivo* antitumour models. As the relationship between duration of drug exposure and cytotoxicity can give valuable information on mechanisms of toxicity (Roed *et al.*, 1986), the patterns of sensitivity to ACLA, ADR, DAU and MITO after short time exposure were compared with the cytotoxicity and cross-resistance patterns after prolonged administration.

Two DAU resistant SCCL cell lines with pronounced differences in expression of the multidrug resistant (MDR) related P-glycoprotein were selected *in vitro* and compared to the parental line.

Correspondence: P.B. Jensen.
Received 31 March 1989; and in revised form 13 July 1989.

### Materials and methods

### *Drugs*

ADR (Carlo Erba), DAU (Rhone Poulenc) and ACLA (Lundbeck) were dissolved in sterile water just before use. MITO (Lederle) was supplied in aqueous solution for infusion. Drug solutions were made with tissue culture medium.

### *Cell lines*

The human SCCL cell lines used were NCI-H69, NCI-N592, NCI-H249CL5, NCI-N417, OC-NYH (also designated GLC-2), OC-TOL (GLC-3), GLC-16 and SCLC-86M1. Their source, relation to therapy and growth behaviour *in vitro* are described in Table I. The serially transplanted mouse tumours Ehrlich ascites tumour and P388 leukaemia were transferred to medium and established as *in vitro* cell lines without changes in DNA content. After three passages *in vitro* the cell lines were used in experiments. Resistance to DAU was obtained by treating a total of approximately $5 \times 10^8$ NCI-H69 cells with $0.1\,\mu g\,ml^{-1}$ of DAU continuously. The surviving cells were pooled and passaged three times in medium with $0.1\,\mu g\,ml^{-1}$ DAU. Cell line NCI-H69 /DAU4 was maintained in drug-free medium for 4 weeks before testing. Cell line NCI-H69/DAU8 was maintained in drug-free medium for 8 weeks before testing.

All cell lines were maintained at 37°C in Roswell Park Memorial Institute medium 1640 with 10% fetal calf serum in a humidified atmosphere with 7.5% $CO_2$. The cell lines were free of mycoplasm contamination, and the genetic stability of the cell lines was checked by flow cytometric DNA-analysis.

### *Clonogenic assay*

Cell survival was assessed by colony formation in soft agar as previously described (Roed *et al.*, 1987). In experiments with 1 h drug exposure the growth conditions before sensitivity testing were standardised, i.e. $5 \times 10^5$ cells were passed to 15 ml of medium and left untouched in flasks for 5 days

**Table I** Source, relation to chemotherapy and growth behaviour *in vitro* of the cell lines used

| *Cell line* | *References* | *Established from* | *Prior therapy* | *Growth behaviour* |
|---|---|---|---|---|
| NCI-H69 | Carney *et al.* (1985) | PlE | CTX MTX CCNU* VCR ADR PRO | s |
| NCI-N592 | Carney *et al.* (1985) | BM | MTX* | s |
| NCI-N417 | Carney *et al.* (1985) | LT | no | s |
| NCI-H249CL5 | Carney *et al.* (1985) | BM | MTX* | s |
| OC-NYH | de Leij *et al.* (1985) | PlE | no | mon |
| OC-TOL | de Leij *et al.* (1985) | PlE | no | s |
| GLC16 | Berendsen *et al.* (1988) | LT | CTX VP-16 ADR | s |
| SCLC-86M1 | Bepler *et al.* (1987) | PlE | no | s |

PlE, pleural effusion; BM, bone marrow; LT, lung tumour; CTX, cyclofosfsmide; MTX, methotrexate; VCR, vincristine; ADR, adriamycin; PRO, procarbazine; s, growth in suspension; mon, growth as monolayer. *D. Carney (personal communication).

before sensitivity testing (Jensen *et al.*, 1989). A single-cell suspension was exposed to each of the drugs for 1 h, washed twice with PBS (150 mM NaCl, 50 mM phosphate, pH 7.2), and plated in soft agar on top of a feeder layer containing sheep red blood cells. After solidification of the agar, 1 ml of medium was added to prevent drying. In each experiment the four drugs were tested on the same batch of cells. The stability of the cytotoxicity of the drugs was assessed in experiments comparing 1-h cytotoxicity of drug solutions preincubated 48 h in tissue culture medium at 37°C with freshly diluted drugs. For continuous incubation, the cells were plated directly in agar with the desired drug concentration. The colonies were counted after three weeks incubation.

*Data analysis*

For each drug the cells were exposed to five concentrations. All experiments were done in triplicate. To obtain linearity on exponential dose-response curves the logarithmically transformed response data were used in linear regression analysis. When a large shoulder was obtained on the dose-response curves only the exponential parts of these curves were used in the analysis. When the intercept of the regression line was below the survival of untreated cells (100% survival) the regression line was recomputed without intercept. The dose reducing the number of colonies to 50% of control ($LD_{50}$) was computed from the regression parameters.

*Flow cytometric DNA-analysis*

Cell samples for flow cytometric DNA-analysis were taken from the single-cell suspensions just before drug exposure. After centrifugation, the cells were suspended in citrate buffer, frozen on ethanol with dry ice and stored at −80°C until analysis (Vindeløv *et al.*, 1983*a*). The samples were stained with propidium iodide (Vindeløv *et al.*, 1983*b*) and analysed in a FACS III flow-cytometer (Becton-Dickinson). The percentage of cells in each cell cycle phase was determined by statistical analysis of the DNA distribution (Christensen *et al.*, 1978).

*Immunological detection of P-glycoprotein*

C219 monoclonal antibody (1 mg $ml^{-1}$) against P-glycoprotein (Kartner *et al.*, 1985) was purchased from Centocor (Malvern, PA, USA). Peroxidase conjugated antibody was purchased from Dakopatts, Copenhagen.

*Western blot* Cells from all human cell lines were spun down at 150 *g* and washed with PBS. The cell pellets were then resuspended (v/v 2:3) in a solution consisting of 3.4 mM sodium citrate, 1.5 mM spermine tetrahydrochloride, 0.5 mM Tris and 1% v/v Nonidet P40 (pH 7.6). After centrifugation at 1,400 *g*, 35 µl of the supernatants were loaded on to a 10% (w/w) SDS-PAGE gel with 0.1% (w/w) SDS. Proteins were blotted on to nitrocellulose paper after electrophoresis. The paper was washed in a 150 mM NaCl, 50 mM Tris (pH 7.4) buffer containing 3% (w/w) bovine serum albumin, and 0.1% (v/v) Tween 20, and probed overnight at 4°C with the C219 antibody at 1:400 dilution. Peroxidase conjugated rabbit anti-mouse antibody (Dako P260) was used as the secondary antibody at a dilution of 1:250 applied for 2 h. The blot was developed using 3-amino-9-ethyl carbazole as chromogen. Controls were performed by omission of the primary antibody.

*Immunohistochemistry* Cell from all human lines were pelleted and frozen sectioned. Frozen sections were fixed in acetone for 15 min. Thereafter a three layer immunohistochemical technique was employed, the first layer being C219 antibody 1:500 overnight at 4°C, the second layer peroxodise conjugated rabbit anti-mouse 1:10 (Dako P260), and the third layer peroxidase conjugated swine anti-rabbit 1:20 (Dako P217) with washes of PBS in between. 3-Amino-9-ethyl carbazole was used as chromogen and Mayer's haematoxylin counterstain. Controls were performed by omission of the primary antibody.

### Results

We have previously shown that the sensitivity of the SCCL cell lines to ADR, DAU and MITO with 1-h incubation is dependent on the size of the S-phase fraction at the time of drug exposure and that reduced variation in the clonogenic assay can be obtained by standardisation of the cell culture conditions (Jensen *et al.*, 1989). In the present study, the inter-experimental variation after continuous incubation was evaluated on cell line NCI-N592 in three experiments with varying S-phase distribution (Table II). Despite a variation with a factor of two in the S-phase distribution, the inter-experimental variation in $LD_{50}$ is less than 25% and not correlated to the size of the S-phase. With similar S-phase variations the sensitivity with 1-h incubation varied 250% (Jensen *et al.*, 1989). Accordingly , standardisation of the growth conditions before sensitivity testing with continuous incubation is not necessary. The results of continuous incubation with ACLA, ADR, DAU and MITO on the eight SCCL lines and the two murine cell lines Ehrlich ascites tumour and P388 leukaemia are shown in Figure 1. For comparison the results with 1-h incubation are depicted in Figure 2. The cell lines are ranked according to the sensitivity obtained with 1-h ADR exposure. As with 1-h incubation, the Ehrlich ascites tumour is the least sensitive cell line to ADR and P388 leukaemia the most sensitive cell line to ADR. Likewise the murine tumours are relatively sensitive to MITO and relatively insensitive to ACLA. This high sensitivity to MITO on the murine tumours is in accordance with previous findings (Hill *et al.*, 1989). Although NCI-H69 is relatively more sensitive to DAU, the sensitivity to ADR and DAU covariate and is almost equal in most cell lines, whereas DAU is more potent than ADR with 1-h incubation. MITO is a potent drug with continuous incubation and the

**Table II** Variation in S-phase distribution and sensitivity to adriamycin, daunorubicin, aclarubicin and mitoxantrone of cell line NCI-N592 in three experiments with continuous incubation.

| | $LD_{50}$ ($\mu g\ ml^{-1}$) | | | |
|---|---|---|---|---|
| *% S phase* | *ADR* | *DAU* | *ACLA* | *MITO* |
| 19 | 0.018 | 0.016 | 0.0058 | 0.0056 |
| 27 | 0.015 | 0.015 | 0.0057 | 0.0058 |
| 34 | 0.016 | 0.015 | 0.0045 | 0.0057 |

relative sensitivity covariates with ADR and DAU. The most striking change from 1-h to continuous drug exposure is the relatively high and uniform potency of ACLA. ACLA is the most potent drug in the three SCCL cell lines least sensitive to ADR. By comparing Figure 1 with Figure 2, only minor changes in the sensitivity pattern of ADR and MITO are seen. Apart from the low ACLA sensitivity of Ehrlich ascites tumour cells, all other cell lines seem relatively sensitive to ACLA with continuous drug exposure. In the human cell

**Figure 1** The sensitivity of eight human SCCL and two murine ascites tumour cell lines to ADR, DAU, ACLA and MITO. Continuous drug exposure. The results are depicted as $LD_{50}$ values. The units on the histograms are calibrated according to the mean $LD_{50}$ of the individual drug to enable the comparison of patterns in sensitivity. Cell lines NCI-H69, NCI-H249CL5, GLC-16 and NCI-N592 were established from treated patients (Table I). EHR (Ehrlich ascites tumour) 69 (NCI-H69), H249 (NCI-H249CL5), TOL (OC-TOL), 592 (NCI-N592), N417 (NCI-N417), NYH (OC-NYH) and 86M1 (SCLC-86M1).

**Figure 2** The sensitivity of eight human small cell and two murine ascites tumour cell lines to ADR, DAU, ACLA and MITO. One-hour drug exposure. The cell lines are ranked according to increasing ADR sensitivity. Observe the increased drug concentrations used compared to Figure 1.

lines, the patterns of 1-h incubation and continuous incubation indicate cross-resistance between ADR, DAU and MITO, whereas the sensitivity pattern to ACLA is different.

With 1-h incubation, the average $LD_{50}$ value obtained with ACLA on the 10 cell lines is $0.39\,\mu g\,ml^{-1}$ and with continuous incubation it was $0.077\,\mu g\,ml^{-1}$. The potency of ACLA is thus increased by a factor of 51. The potency of MITO, ADR and DAU are increased by factors of only 16, 13 and 6 respectively.

The sensitivity of the DAU resistant cell lines NCI-H69/DAU4 and NCI-H69/DAU8 was assessed with continuous incubation (Figure 3). Compared to the parental line, the resistant cell lines are cross-resistant to ADR and partially resistant to MITO and ACLA. Resistance to anthracyclines has been attributed to a 'leak and pump' model of the cell membrane with an active efflux of the anthracyclines proposed to be linked to a glycoprotein (P-glycoprotein) in the cell membrane (Bradley *et al.*, 1988). Western blot analysis with the monoclonal antibody C-219 directed against the P-glycoprotein was performed on all the human cell lines. There was no detectable staining for P-glycoprotein on the wild type lines (not shown). In the resistant lines there was barely detectable glycoprotein on NCI-H69/DAU8 whereas the glycoprotein was clearly discernible on cell line NCI-H69/DAU4 (Figure 4). Based on the dilution shown in Figure 4 the limit of detection is between 5 and 10% of the amount seen in NCI-H69/DAU4, and it is seen that NCI-H69/DAU8 has less than 10% of the P-glycoprotein found in NCI-H69/DAU4. Likewise the use of immunohistochemistry did not recognise P-glycoprotein on the wild type cell lines or on NCI-H69/DAU8 and confirmed the Western blot detection of P-glycoprotein in NCI-H69/DAU4 (not shown).

### Discussion

In this panel of eight SCCL cell lines established from treated and untreated patients the sensitivities to the four drugs vary within a factor of only five. Thus, a minimal inter-experimental variation is necessary in establishing sensitivity profiles from a panel of cell lines. With short time incubation, the variation is mainly due to variations in the S-phase fraction. Consequently it is necessary to standardise growth conditions before sensitivity testing to reduce the variation in the S-phase fraction. The present investigation has shown that the inter-experimental variation with continuous incubation is small and not correlated to the size of the S-phase fraction at the start of the incubation. This finding is in accordance with the longer drug exposure time, ensuring that all cells pass through sensitive cell-cycle phases.

The almost identical sensitivity patterns obtained after 1-h incubation and continuous incubation strongly support the value of continuous incubation. A limited drug stability leading to poorly defined concentration–time products is, however, an obvious problem in interpreting data from experiments with continuous incubation. There seem to be no major differences in the stability of DAU, ACLA and MITO is physiological solutions. Thus DAU, ACLA and MITO are 95% stable in neutral 150 mM NaCl solutions at room temperature for 40, 52 and more than 48 h respectively (Bosanquet, 1986). As shown by Bosanquet, no consensus exists in the literature regarding the stability of ADR, which has been most extensively studied, and it is suggested that similar discrepancies would be found if the other drugs were investigated as intensively. Therefore we tested the stability of the cytotoxicity of DAU and ACLA by comparing the

**Figure 3** Dose–response curves obtained with continuous incubation of ACLA (**a**), ADR (**b**), DAU (**c**) and MITO (**d**) on the DAU-resistant sublines NCI-H69/DAU4 (------) and NCI-H69/DAU8 (——) compared to the parental cell line NCI-H69 (———). Regression analysis gave the following $LD_{50}$ values for: DAU, 0.011, 0.047 and $0.105\,\mu g\,ml^{-1}$; ACLA, 0.0050, 0.014 and $0.019\,\mu g\,ml^{-1}$ on NCI-H69, NCI-H69/DAU8, and NCI-H69/DAU4 respectively.

**Figure 4** Western blot detection of P-glycoprotein with C219 monoclonal antibody. 1, P388 sensitive and, 2, P388/DAU + daunorubicin resistant murine controls. In the sensitive line C219 binds to protein with a lower molecular weight than P-glycoprotein; 3, the parental cell line NCI-H69; 4, subline NCI-H69/DAU8; 5, subline NCI-H69/DAU4; 6, 75%; 7, 50%; 8, 25%; 9, 10%; 10, 5% dilution of the protein loaded to lane 5; 11, Ehrlich/DAU + daunorubicin resistant and 12, wild type Ehrlich ascites tumour murine controls.

activity of freshly diluted drugs with drugs preincubated for 48 h at 37°C in medium. In two experiments on cell lines NCI-H69 and NCI-N592 with 1-h drug exposure we found a 20 and 25% increase in the $LD_{50}$ value of ACLA and a 3 and 5% increase in the $LD_{50}$ value of preincubated DAU. Thus the relative increase in potency with continuous incubation cannot be explained by a higher stability of ACLA than of DAU. A possible explanation of the increase in potency elicited by ACLA with prolonged exposure time could be that ACLA preferentially inhibit RNA synthesis, especially nucleolar RNA synthesis (Skovsgaard, 1987; Oki *et al.*, 1981), in contrast to ADR and DAU, which inhibit DNA- and RNA-synthesis to almost the same degree.

*In vitro* resistance and cross-resistance to ACLA have been studied in a number of anthracycline-resistant murine and human tumour cell lines (Table III). Development of resistance to ACLA in the L5178Y cell line was accompanied by a reduction in the sensitivity to DAU and ADR (Nishimura *et al.*, 1980). However, only minimal cross-resistance was found in a range of human and murine cancer cell lines highly resistant to DAU and ADR (Nishimura *et al.*, 1978; Hill *et al.*, 1985; Umezawa *et al.*, 1987; Scott *et al.*, 1986; Twentyman *et al.*, 1986) (Table III). *In vivo*, ACLA was found to be effective in P388 resistant to m-AMSA (Johnson & Howard, 1982) and some activity was reported on P388 resistant to ADR (Oki *et al.*, 1981). In contrast, ACLA was ineffective *in vivo* against Ehrlich ascites tumour resistant to DAU (Skovsgaard, 1987). We have compared the DAU resistant subline of Ehrlich ascites tumour to the wild type *in vitro*. The $LD_{50}$ of ACLA was increased by a factor of 2.3 whereas a 20-fold increase was found with DAU. Since the Ehrlich wild type is not very sensitive to ACLA, such a low decrease in ACLA sensitivity is sufficient to result in total loss of activity. The sublines NCI-N69/DAU4 and NCI-H69/DAU8 are cross-resistant not only to ADR and MITO, but also to ACLA. However, as seen in Figure 3, both H69/DAU8 and H69/DAU4 are more sensitive to ACLA than to

**Table III** Relative resistance in drug resistant cancer cell lines

| | Cell | | Resistant | *In vitro* resistance factor[a] | | | *In vivo* | | |
|---|---|---|---|---|---|---|---|---|---|
| *Reference* | *line* | *Type* | *to* | *ACLA* | *DAU* | *ADR* | *ACLA* | *DAU* | *ADR* |
| Nishimura *et al.* (1980) | L5178Y | MUR | ACLA | 11 | 27 | 42 | | | |
| Nishimura *et al.* (1978) | – | – | ADR | 2 | 20 | 20 | | | |
| Hill *et al.* (1985) | – | – | ADR | 1.2 | 2.5 | 2.5 | | | |
| Umezawa *et al.* (1987) | P388 | – | ADR | 1.6 | 22 | 26 | | | |
| Scott *et al.* (1986) | – | – | ADR | 1.4 | – | 14 | | | |
| Johnson & Howard (1982) | – | – | M-AMSA | – | – | – | SEN | PR | RES |
| Oki *et al.* (1981) | – | – | ADR | – | – | – | PR | | |
| Skovsgaard (1987) | EHRLICH | – | DAU | 2.3[b] | 20[b] | 60[b] | RES | RES | |
| Scott *et al.* (1986) | CCRF CEM | HUM | ADR | 1.2 | – | 5.7 | | | |
| Scott *et al.* (1986) | U266BL | – | ADR | 1.2 | – | 9.3 | | | |
| Twentyman *et al.* (1986) | NCI-H69 | – | ADR | 0.8–1.1 | – | 5–25 | | | |
| Twentyman *et al.* (1986) | – | – | ADR | 2.3 | – | 50 | | | |
| | – | – | DAU | 2.9[c] | 4.4[c] | – | | | |
| | – | – | DAU | 3.8[c] | 9.4[c] | – | | | |

MUR, murine; HUM, human; SEN, sensitive; PR, partially resistant; RES, resistant. [a]The *in vitro* resistance factor is the ratio between doses giving the same effect in the resisted cell line and in the parent cell line. [b]E. Friche (unpublished results). [c]Results from the present investigation.

ADR or DAU. Although accurate $LD_{50}$ values cannot be determined because of the limited cell kill obtained with DAU in the resistant cell lines, it is clear that the relative resistance to DAU is more pronounced than to ACLA and MITO. Thus these results and the results summarised in Table III all suggest limited cross-resistance to ACLA in tumour cell lines made resistant to ADR or DAU. As the parental cell line NCI-H69 was obtained from a patient who had been heavily pretreated with ADR (Table I) the clinical relevance of the increased resistance of the sublines may be limited. No P-glycoprotein was detected on the wild type lines, whereas the protein was abundant in NCI-H66/DAU4. The ACLA sensitivity of NCI-H69/DAU8, with 10% or less P-glycoprotein content compared to NCI-H69/DAU4, is similar to the ACLA sensitivity of NCI-H69/DAU4. Thus there seems to be no linear relationship between the amount of P-glycoprotein and the resistance to ACLA. In this context it is interesting that only a modest reduction of ACLA accumulation has been found in cell lines resistant to ACLA (Skovsgaard, 1987).

The clinical results of ACLA treatment in solid tumours have been disappointing so far. The drug has not shown activity against a range of tumours (Aabo *et al.*, 1983; Woolley *et al.*, 1982; Kerpel-Fronius *et al.*, 1987) including SCCL (Lev & Posada, 1983; Kramer *et al.*, 1986; Abeloff *et al.*, 1985). The doses have invariably been significantly lower in the solid tumour trials (e.g. $100\ \mathrm{mg\ m^{-2}}$ as a bolus treatment) compared to the high doses of ACLA that have been used in leukaemia trials (e.g. $80\ \mathrm{mg\ m^{-2}}$ for three consecutive days). In this context it has been demonstrated (Machover *et al.*, 1984) that the cumulative dose required for the majority of previously treated leukaemia patients to achieve complete remission is $>300\ \mathrm{mg\ m^{-2}}$. Machover *et al.* (1984) used 10-day courses of ACLA at a daily dose of $15\ \mathrm{mg\ m^{-2}}$ with 10-day intervals between courses. This regimen was given to 25 patients with AML who were either refractory to initial induction chemotherapy or in relapse. Eleven patients (44%) achieved complete remission. These results are comparable to the results obtained with shorter schedules using considerably higher doses. Although optimal schedules cannot be determined *in vitro*, the present study also indicates a relative increase in ACLA potency with prolonged administration. However, the importance of a prolonged schedule still remains to be determined in a randomised trial.

If sensitivity patterns on cell lines obtained from treated and untreated patients with SCCL give a reliable picture of the disease, the lack of ACLA resistance on the panel of SCCL cell lines and the potency of ACLA seen with prolonged administration justify new clinical trials of ACLA against SCCL. In favour of this approach is also the great difficulty in obtaining *in vitro* resistance to ACLA that has been described from different groups. Tapiero *et al.* (1988) took 3 years to obtain a 6-fold ACLA resistant cell line, whereas a 100-fold ADR resistant cell line was obtained in a month. Likewise, Nishimura *et al.* (1980) tried without success to isolate ACLA resistant cells *in vitro*. We have recently initiated a phase II trial of ACLA on patients with relapse of SCCL using the high leukaemia doses in a three day schedule as this schedule has been most thoroughly tested.

Eva Høj and Annette Nielsen are thanked for excellent technical assistance, and John Post for preparing the manuscript. Supported by grants from the Danish Cancer Society and from the Lundbeck foundation.

### References

AABO, K., MORTENSEN, S.Å., SKOVSGAARD, T. & GYEMOSE, E. (1983). Intermittent high-dose aclarubicin in patients with advanced cancer: a phase I study with special reference to cardiac toxicity. *Cancer Treat. Rep.*, **67**, 281.

ABELOFF, M.D., FINKELSTEIN, D.M., CHANG, A.Y.C., CAMACHO, F.J., CREECH, H. & ETTINGER, D.S. (1985). Phase II study of aclarubicin and diaziquone in the treatment of advanced small cell bronchogenic carcinoma (EST 4581): an Eastern Cooperative Oncology Group Study. *Cancer Treat. Rep.*, **69**, 451.

BEPLER, G., JAQUES, G., NEUMANN, K. & 3 others (1987). Establishment, growth properties, and morphological characteristics of permanent human small cell lung cancer cell lines. *J. Cancer Clin. Oncol.*, **113**, 31.

BERENDSEN, H.H., DE LEIJ, L.F.M.H., DE VRIES, E.G.E. & 7 others (1988). Characterization of three small cell lung cancer cell lines established from one patient during longitudinal follow-up. In *Search for the Therapy Resistant Phenotype in Lung Cancer*, (ed) p. 33. Thesis, Groningen.

BOSANQUET, A.G. (1986). Stability of solutions of antineoplastic agents during preparation and storage for in vitro assays. II. Assay methods, adriamycin and the other antitumor antibiotics. *Cancer Chemother. Pharmacol.*, **17**, 1.

BRADLEY, G., JURANKA, P.F. & LING, V. (1988). Mechanism of multidrug resistance. *Biochim. Biophys. Acta*, **948**, 87.

CARNEY, D.N., GAZDAR, A.F., BEPLER, G. & 5 others (1985). Establishment and identification of small cell lung cancer cell lines having classic and variant features. *Cancer Res.*, **45**, 2913.

CHRISTENSEN, I., HARTMAN, N.R., KEIDING, N., LARSEN, J.K., NOER, H. & VINDELØV, L. (1978). Statistical analysis of DNA distributions from cell populations with partial synchrony. In *Pulsecytophotometry, Part 3*, Lutz, D. (ed) p. 71. European Press Medicon: Ghent.

DE LEIJ, L., POSTMUS, P.E., BUYS, C.H.C.M. & 7 others (1985). Characterization of three new variant type cell lines derived from small cell carcinoma of the lung. *Cancer Res.*, **45**, 6024.

HANSEN, O.P., ELLEGAARD, J., MADSEN, P.B. & 14 others (1988). Combination chemotherapy with aclarubicin plus cytosine arabinoside versus daunorubicin plus cytosine arabinoside in denovo acute myelocytic leukemia (AML): a Danish national trial. *Proc. ASCO*, **7**, 175.

HILL, B.T., DENNIS, L.Y., LI, X.T. & WHELAN, R.D.H. (1985). Identification of anthracycline analogues with enhanced cytotoxicity and lack of cross-resistance to adriamycin using series of mammalian cell lines *in vitro*. *Cancer Chemother. Pharmacol.*, **14**, 194.

HILL, B.T., HOSKING, L.K., SHELLARD, S.A. & WHELAN, R.D.H. (1989). Comparative effectiveness of mitoxantrone and doxorubicin in overcoming experimentally induced drug resistance in murine and human tumour cell lines *in vitro*. *Cancer Chemother. Pharmacol.*, **23**, 140.

JENSEN, P.B., ROED, H., VINDELØV, L., CHRISTENSEN, I.J. & HANSEN, H.H. (1989). Reduced variation in the clonogenic assay obtained by standardization of the cell culture conditions prior to drug testing on human small cell lung cancer cell lines. *Invest. New Drugs* (in the press).

JOHNSON, R.K. & HOWARD, W.S. (1982). Development and cross-resistant characteristics of a subline of P388 leukemia resistant to 4'-(9-acridinylamino)-methanesulfon-*m*-anisidide. *Eur. J. Cancer Clin. Oncol.*, **18**, 479.

KARTNER, N., EVERNDEN-PORELLE, D., BRADLEY, G. & LING, V. (1985). Detection of P-glycoprotein in multidrug-resistant cell lines by monoclonal antibodies. *Nature*, **316**, 820.

KERPEL-FRONIUS, S., GYERGYAY, F., HINDY, I. & 9 others (1987). Phase I-II trial of aclacinomycin A given in a four-consecutive-day schedule to patients with solid tumors. *Oncology*, **44**, 159.

KRAMER, B.S., BIRCH, R., GOCKERMAN, J.P., GRECO, A. & PRESTRIDGE, K. (1986). Phase II evaluation of aclarubicin in lung cancer: a Southeastern Cancer Study Group Trial. *Cancer Treat. Rep.*, **70**, 803.

LEV, L.M. & POSADA, J.G. JR. (1983). Aclarubicin: status of phase I and II studies in the United States. *Cancer Treat. Symp.*, **1**, 65.

MACHOVER, D., GASTIABURU, J., DELGADO, M. & 8 others (1984). Phase I-II study of aclarubicin for treatment of acute myeloid leukemia. *Cancer Treat. Rep.*, **68**, 881.

NISHIMURA, T., MUTO, K. & TANAKA, N. (1978). Drug sensitivity of an adriamycin-resistant mutant subline of mouse lymphoblastoma L5178Y cells. *J. Antibiot.*, **31**, 493.

NISHIMURA, T., SUZUKI, H., MUTO, K., TANAKA, Y. & TANAKA, N. (1980). Studies on aclacinomycin A resistance in a mouse lymphoblastoma. *J. Antibiot.*, **33**, 737.

OKI, T., MATSUZAWA, Y., YOSHIMOTO, A. & 10 others (1975). New antitumor antibiotics, aclacinomycins A and B. *J. Antibiot.*, **28**, 830.

OKI, T., TAKEUCHI, T., OKA, S. & UMEZAWA, H. (1981). New anthracycline antibiotic aclacinomycin A: experimental studies and correlations with clinical trials. *Rec. Results Cancer Res.*, **76**, 21.

PEDERSEN-BJERGAARD, J., BRINCKER, H., ELLEGAARD, J. 5 others (1984). Aclarubicin in the treatment of acute nonlymphocytic leukemia refractory to treatment with daunomycin and cytarabine: a phase II trial. *Cancer Treat. Rep.*, **68**, 1233.

ROED, H., CHRISTENSEN, I.J., VINDELØV, L.L., SPANG-THOMSEN, M. & HANSEN, H.H. (1987). Inter-experiment variation and dependence on culture conditions in assaying chemosensitivity of human small cell lung cancer lines. *Eur. J. Cancer Clin. Oncol.*, **23**, 177.

ROED, H., VINDELØV, L.L., SPANG-THOMSEN, M. & ENGELHOLM, S.AA. (1986). Limitations and potentials of in vitro sensitivity testing of human small cell carcinoma of the lung. In *Lung Cancer: Basic and Clinical Aspects,* Hansen, H.H. (ed), p. 77. Martinus Nijhoff: Boston.

SCOTT, C.A., WESTMACOTT, D., BROADHURST, M.J., THOMAS, G.J. & HALL, M.J. (1986). 9-Alkyl anthracyclines. Absence of cross-resistance to adriamycin in human and murine cell cultures. *Br. J. Cancer,* **53**, 595.

SKOVSGAARD, T. (1987). Pharmacodynamic aspects of aclarubicin with special reference to daunorubicin and doxorubicin. *Eur. J. Haematol.*, **38**, 7.

TAPIERO, H., BOULE, D., ITTAH-CORCOS, N. & FOURCADE, A. (1988). Manipulation of drug accumulation: Mechanisms to overcome resistance. In *Mechanisms of Drug Resistance in Neoplastic Cells,* Woolley, P.V. & Tew, K.D. (eds) p. 329. Academic Press: San Diego.

TWENTYMAN, P.R., FOX, N.E., WRIGHT, K.A. & BLEEHEN, N.M. (1986). Derivation and preliminary characterization of adriamycin resistant lines of human lung cancer cells. *Br. J. Cancer,* **53**, 529.

UMEZAWA, K., KUNIMOTO, S. & TAKEUCHI, T. (1987). Experimental studies of new anthracyclines: aclacinomycin, THP-adriamycin and ditrisarubicins. *Biomed. Pharmacother.*, **41**, 206.

VINDELØV, L.L., CHRISTENSEN, I.J., KEIDING, N., SPANG-THOMSEN, M., & NISSEN, N.I. (1983*a*) Long-term storage of samples for flow-cytometric DNA analysis. *Cytometry,* **3**, 317.

VINDELØV, L.L., CHRISTENSEN, I.J., & NISSEN, N.I. (1983*b*). A detergent-trypsin method for the preparation of nuclei for flow cytometric DNA analysis. *Cytometry,* **3**, 323.

WARRELL, R.P., ARLIN, Z.A., KEMPIN, S.J. & YOUNG, C.W. (1982). Phase I-II evaluation of a new antracycline antibiotic, aclacinomycin A, in adults with refractory leukemia. *Cancer Treat. Rep.*, **66**, 1619.

WOOLLEY. P.V., AYOOB, M.J., LEVENSON, S.M. & SMITH, F.P. (1982). A phase I clinical trial of aclacinomycin A administered on a five-consecutive-day schedule. *J. Clin. Pharmacol.*, **22**, 359.


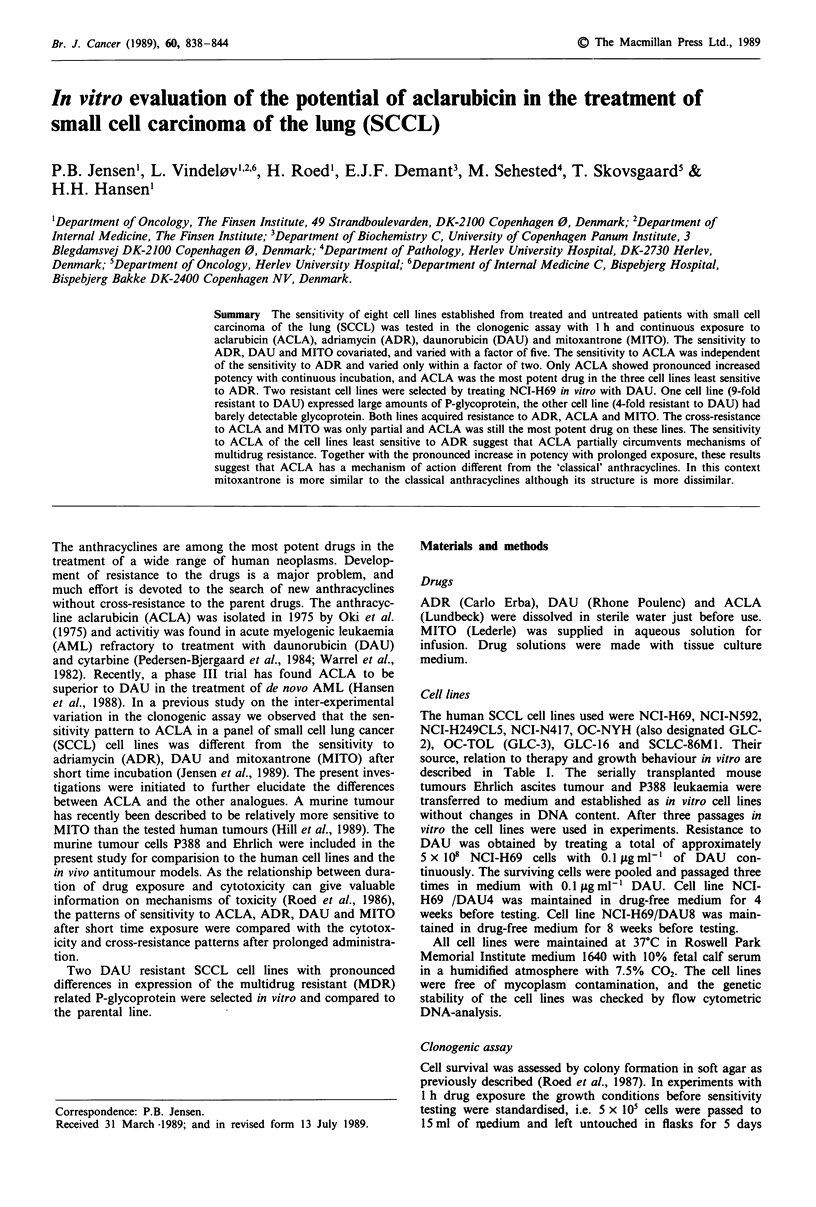

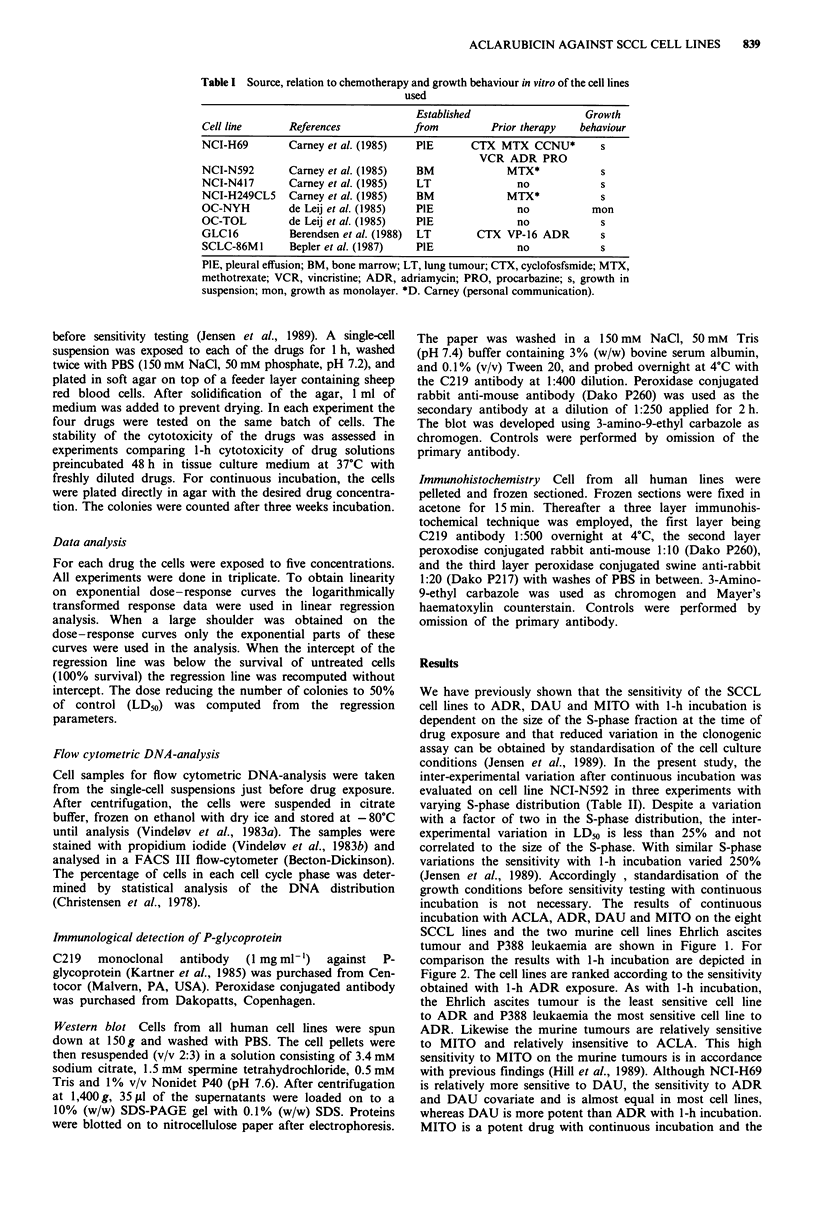

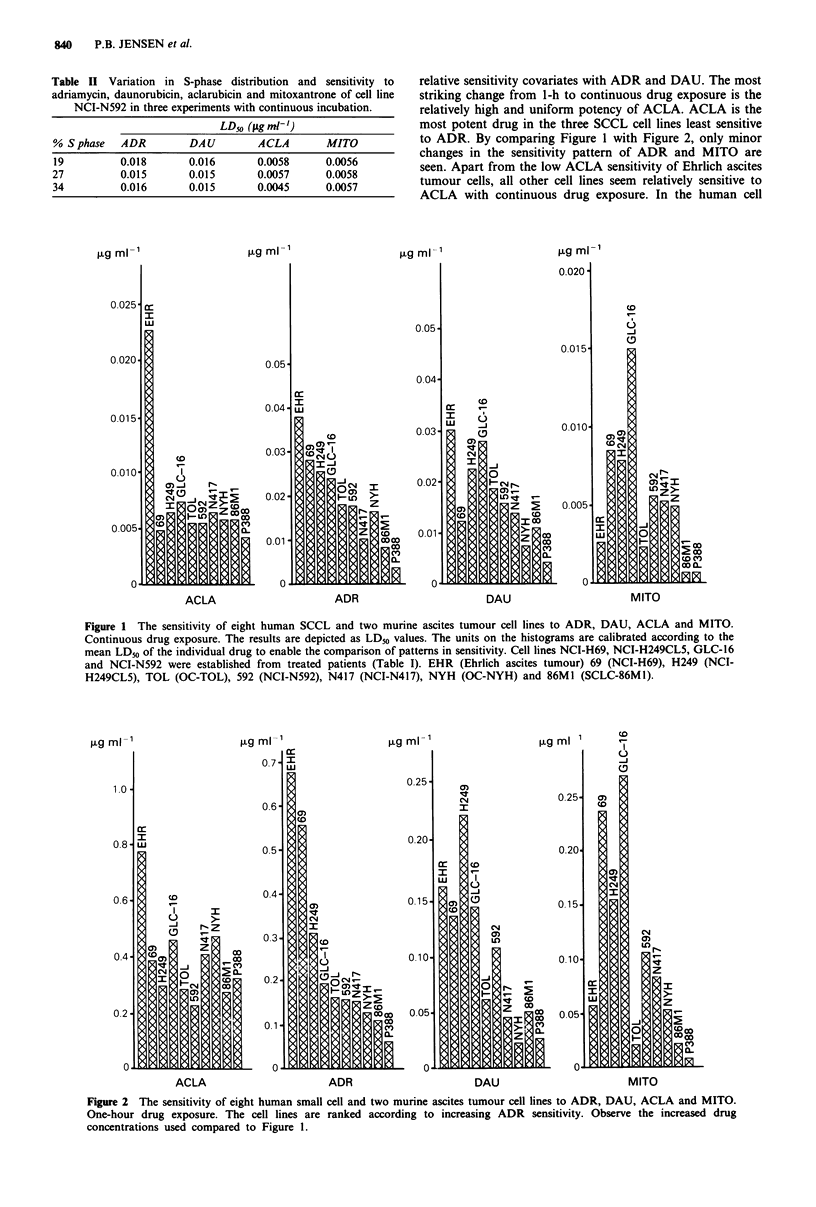

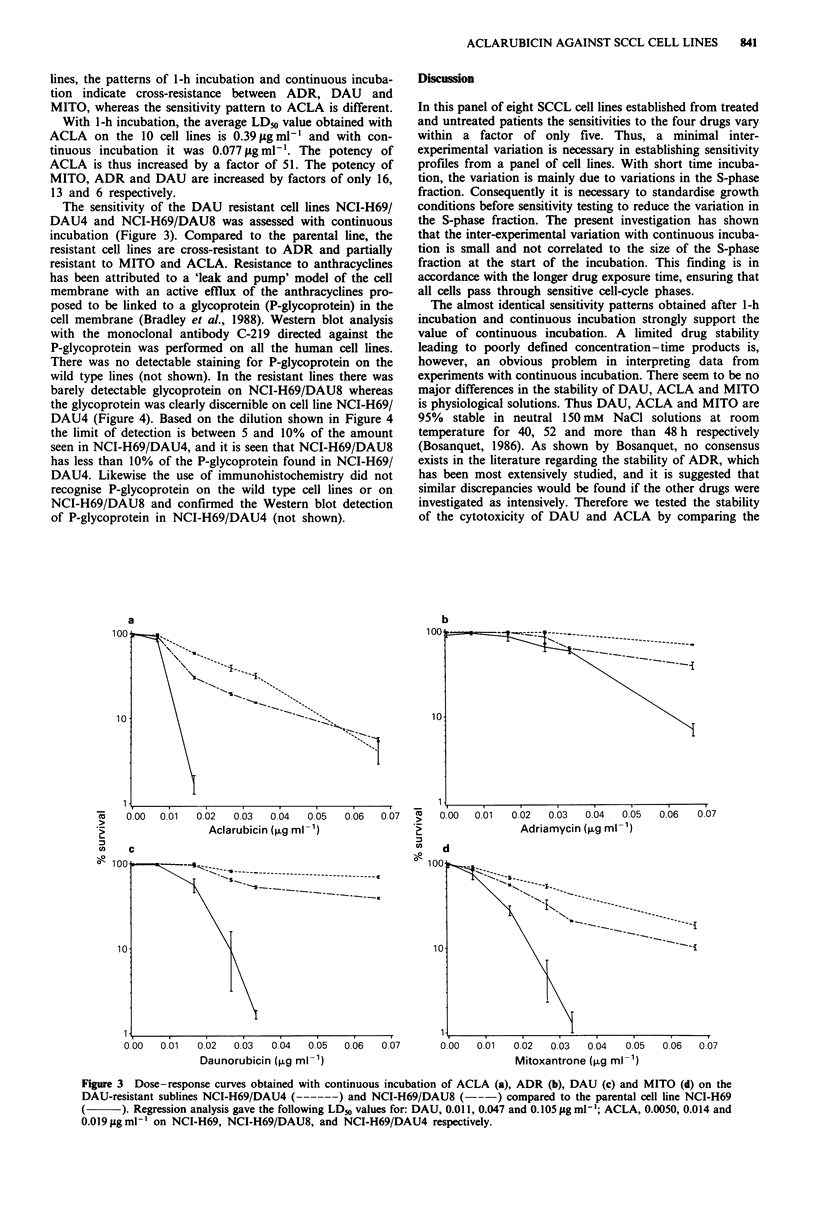

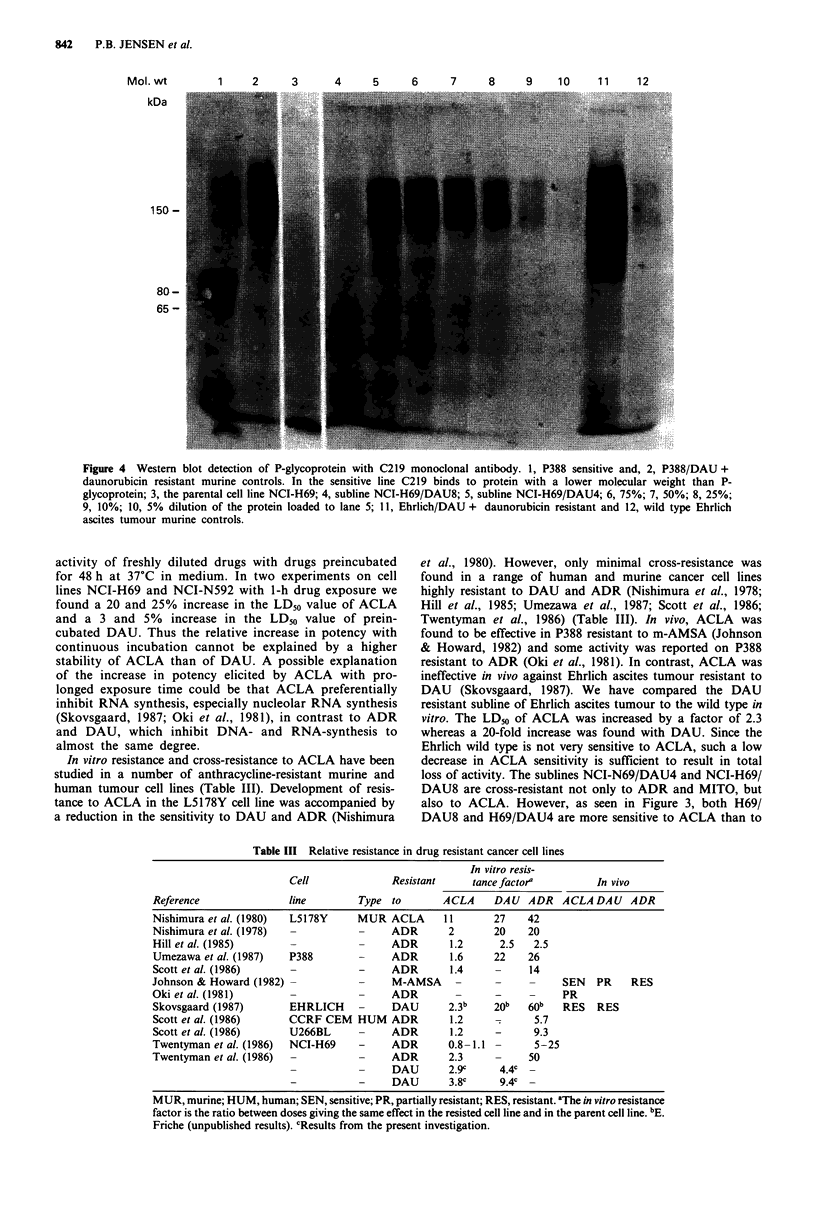

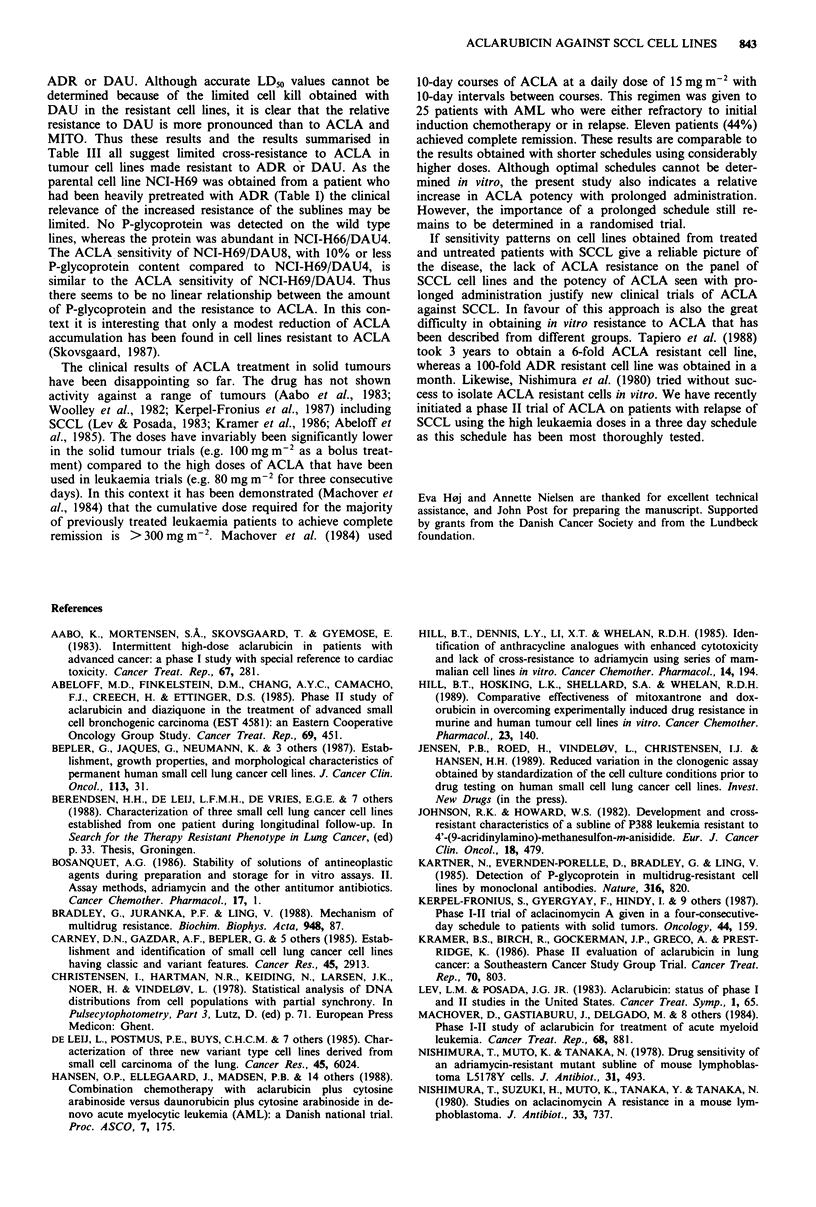

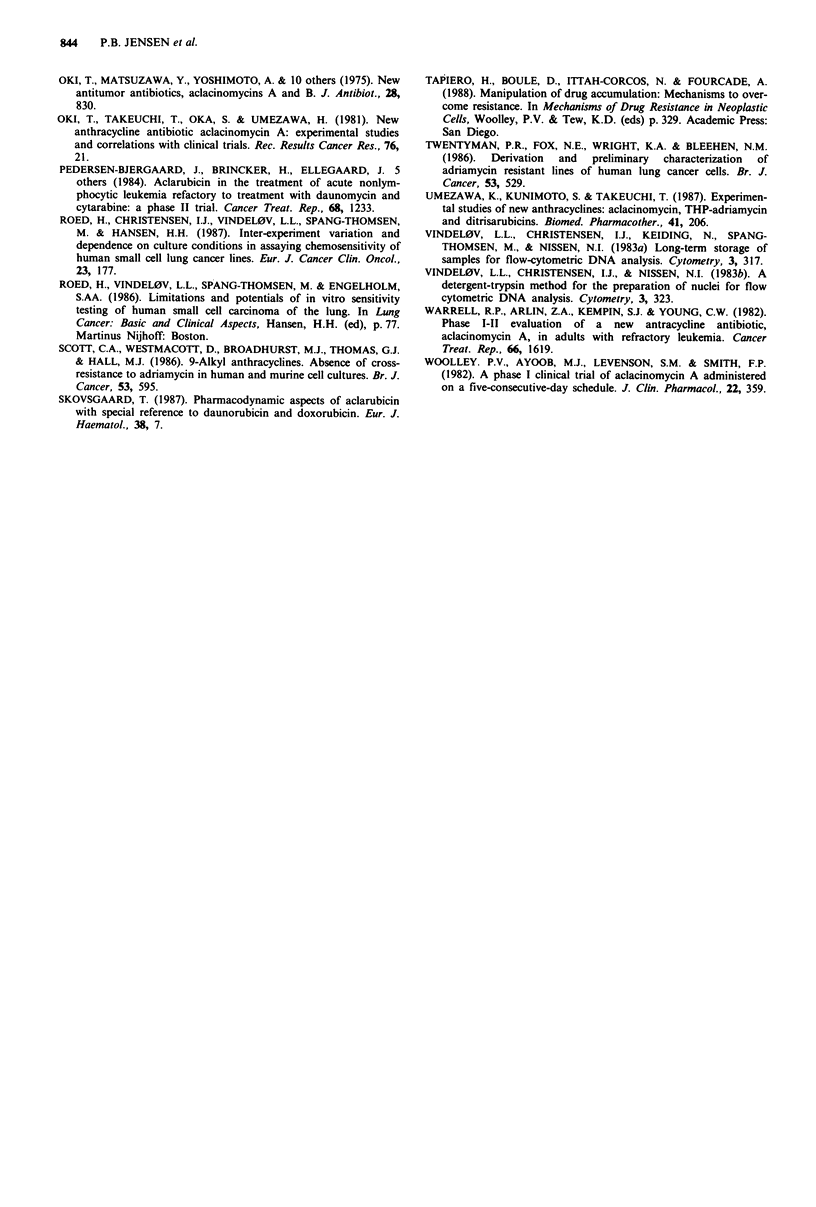


## References

[OCR_00666] Aabo K., Mortensen S. A., Skovsgaard T., Gymoese E. (1983). Intermittent high-dose aclarubicin in patients with advanced cancer: a phase I study with special reference to cardiac toxicity.. Cancer Treat Rep.

[OCR_00672] Abeloff M. D., Finkelstein D. M., Chang A. Y., Camacho F. J., Creech R. H., Ettinger D. S. (1985). Phase II study of aclarubicin and diaziquone in the treatment of advanced small cell bronchogenic carcinoma (EST 4581): an Eastern Cooperative Oncology Group Study.. Cancer Treat Rep.

[OCR_00692] Bosanquet A. G. (1986). Stability of solutions of antineoplastic agents during preparation and storage for in vitro assays. II. Assay methods, adriamycin and the other antitumour antibiotics.. Cancer Chemother Pharmacol.

[OCR_00698] Bradley G., Juranka P. F., Ling V. (1988). Mechanism of multidrug resistance.. Biochim Biophys Acta.

[OCR_00702] Carney D. N., Gazdar A. F., Bepler G., Guccion J. G., Marangos P. J., Moody T. W., Zweig M. H., Minna J. D. (1985). Establishment and identification of small cell lung cancer cell lines having classic and variant features.. Cancer Res.

[OCR_00726] Hill B. T., Dennis L. Y., Li X. T., Whelan R. D. (1985). Identification of anthracycline analogues with enhanced cytotoxicity and lack of cross-resistance to adriamycin using a series of mammalian cell lines in vitro.. Cancer Chemother Pharmacol.

[OCR_00731] Hill B. T., Hosking L. K., Shellard S. A., Whelan R. D. (1989). Comparative effectiveness of mitoxantrone and doxorubicin in overcoming experimentally induced drug resistance in murine and human tumour cell lines in vitro.. Cancer Chemother Pharmacol.

[OCR_00745] Johnson R. K., Howard W. S. (1982). Development and cross-resistance characteristics of a subline of P388 leukemia resistant to 4'-(9-acridinylamino)-methanesulfon-m-anisidide.. Eur J Cancer Clin Oncol.

[OCR_00751] Kartner N., Evernden-Porelle D., Bradley G., Ling V. Detection of P-glycoprotein in multidrug-resistant cell lines by monoclonal antibodies.. Nature.

[OCR_00756] Kerpel-Fronius S., Gyergyay F., Hindy I., Decker A., Sawinsky I., Fäller K., Mechl Z., Nekulova M., Kolaric K., Tomek R. (1987). Phase I-II trial of aclacinomycin A given in a four-consecutive-day schedule to patients with solid tumours. A South-East European Oncology Group (SEEOG) Study.. Oncology.

[OCR_00762] Kramer B. S., Birch R., Gockerman J. P., Greco A., Prestridge K. (1986). Phase II evaluation of aclarubicin in lung cancer: a Southeastern Cancer Study Group Trial.. Cancer Treat Rep.

[OCR_00769] Machover D., Gastiaburu J., Delgado M., Goldschmidt E., Hulhoven R., Misset J. L., de Vassal F., Tapiero H., Ribaud P., Schwarzenberg L. (1984). Phase I-II study of aclarubicin for treatment of acute myeloid leukemia.. Cancer Treat Rep.

[OCR_00774] Nishimura T., Muto K., Tanaka N. (1978). Drug sensitivity of an adriamycin-resistant mutant subline of mouse lymphoblastoma L5178Y cells.. J Antibiot (Tokyo).

[OCR_00787] Oki T., Matsuzawa Y., Yoshimoto A., Numata K., Kitamura I. (1975). New antitumor antibiotics aclacinomycins A and B.. J Antibiot (Tokyo).

[OCR_00792] Oki T., Takeuchi T., Oka S., Umezawa H. (1981). New anthracycline antibiotic aclacinomycin A: experimental studies and correlations with clinical trials.. Recent Results Cancer Res.

[OCR_00798] Pedersen-Bjergaard J., Brincker H., Ellegaard J., Drivsholm A., Freund L., Jensen K. B., Jensen M. K., Nissen N. I. (1984). Aclarubicin in the treatment of acute nonlymphocytic leukemia refractory to treatment with daunorubicin and cytarabine: a phase II trial.. Cancer Treat Rep.

[OCR_00804] Roed H., Christensen I. B., Vindeløv L. L., Spang-Thomsen M., Hansen H. H. (1987). Inter-experiment variation and dependence on culture conditions in assaying the chemosensitivity of human small cell lung cancer cell lines.. Eur J Cancer Clin Oncol.

[OCR_00818] Scott C. A., Westmacott D., Broadhurst M. J., Thomas G. J., Hall M. J. (1986). 9-alkyl anthracyclines. Absence of cross-resistance to adriamycin in human and murine cell cultures.. Br J Cancer.

[OCR_00824] Skovsgaard T. (1987). Pharmacodynamic aspects of aclarubicin with special reference to daunorubicin and doxorubicin.. Eur J Haematol Suppl.

[OCR_00836] Twentyman P. R., Fox N. E., Wright K. A., Bleehen N. M. (1986). Derivation and preliminary characterisation of adriamycin resistant lines of human lung cancer cells.. Br J Cancer.

[OCR_00842] Umezawa K., Kunimoto S., Takeuchi T. (1987). Experimental studies of new anthracyclines: aclacinomycin, THP-adriamycin and ditrisarubicins.. Biomed Pharmacother.

[OCR_00847] Vindeløv L. L., Christensen I. J., Keiding N., Spang-Thomsen M., Nissen N. I. (1983). Long-term storage of samples for flow cytometric DNA analysis.. Cytometry.

[OCR_00851] Vindeløv L. L., Christensen I. J., Nissen N. I. (1983). A detergent-trypsin method for the preparation of nuclei for flow cytometric DNA analysis.. Cytometry.

[OCR_00856] Warrell R. P., Arlin Z. A., Kempin S. J., Young C. W. (1982). Phase I--II evaluation of a new anthracycline antibiotic, aclacinomycin A, in adults with refractory leukemia.. Cancer Treat Rep.

[OCR_00862] Woolley P. V., Ayoob M. J., Levenson S. M., Smith F. P. (1982). A phase I clinical trial of aclacinomycin A administered on a five-consecutive-day schedule.. J Clin Pharmacol.

[OCR_00714] de Leij L., Postmus P. E., Buys C. H., Elema J. D., Ramaekers F., Poppema S., Brouwer M., van der Veen A. Y., Mesander G., The T. H. (1985). Characterization of three new variant type cell lines derived from small cell carcinoma of the lung.. Cancer Res.

